# Supplementary material for: Differential cognitive and clinical improvements in Schizophrenia and bipolar disorder following hospitalization: A comparative analysis based on the Clock Drawing Test
Source: PLoS One. 2025 Jul 8;20(7):e0325537. doi: 10.1371/journal.pone.0325537 (PMC12237019; doi:10.1371/journal.pone.0325537)
Supplement: Attachment — Submitted filename: illustration-eg.pdf [file pone.0325537.s002.pdf]

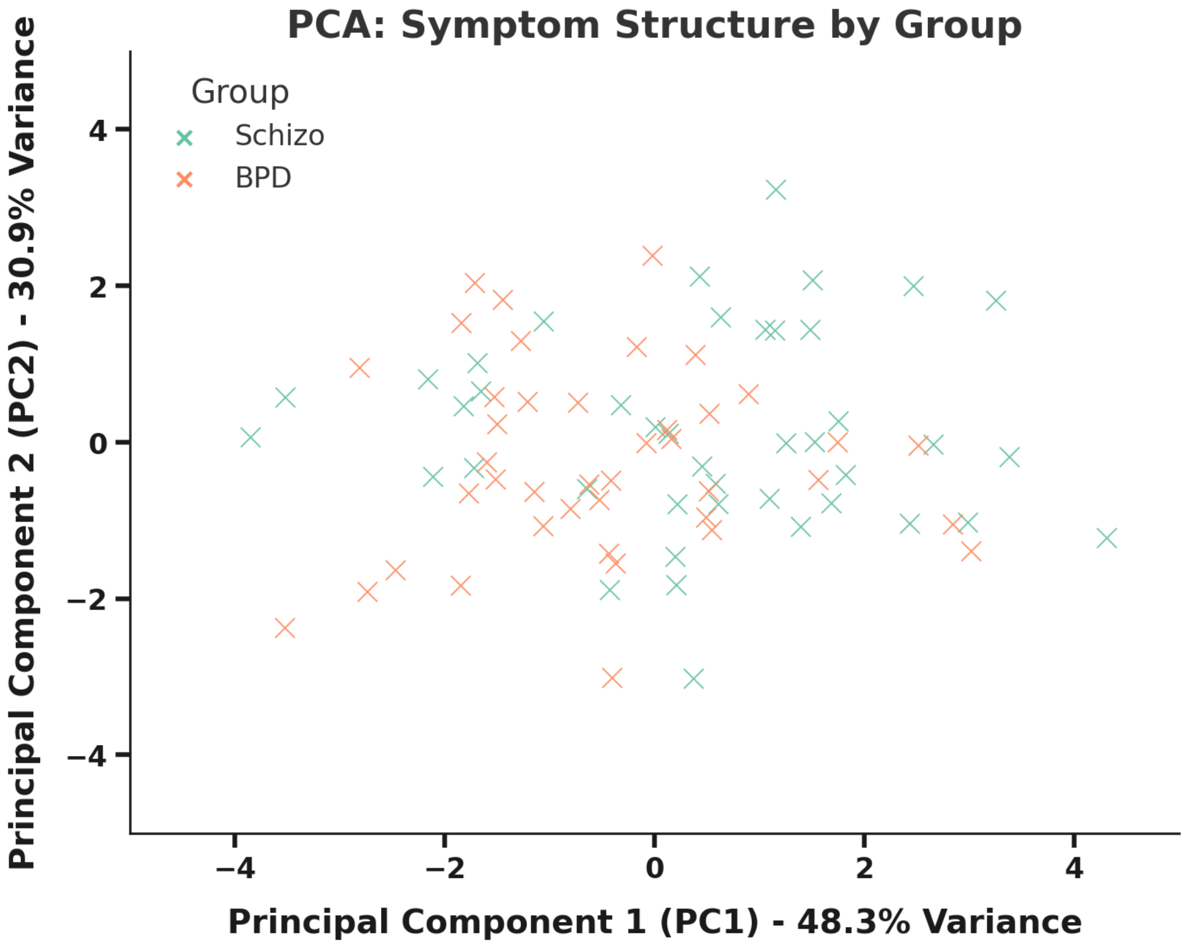

PCA: Symptom Structure by Group
Group
Schizo
BPD
Principal Component 2 (PC2) - 30.9% Variance
Principal Component 1 (PC1) - 48.3% Variance
4
2
0
−2
−4
−4
−2
0
2
4

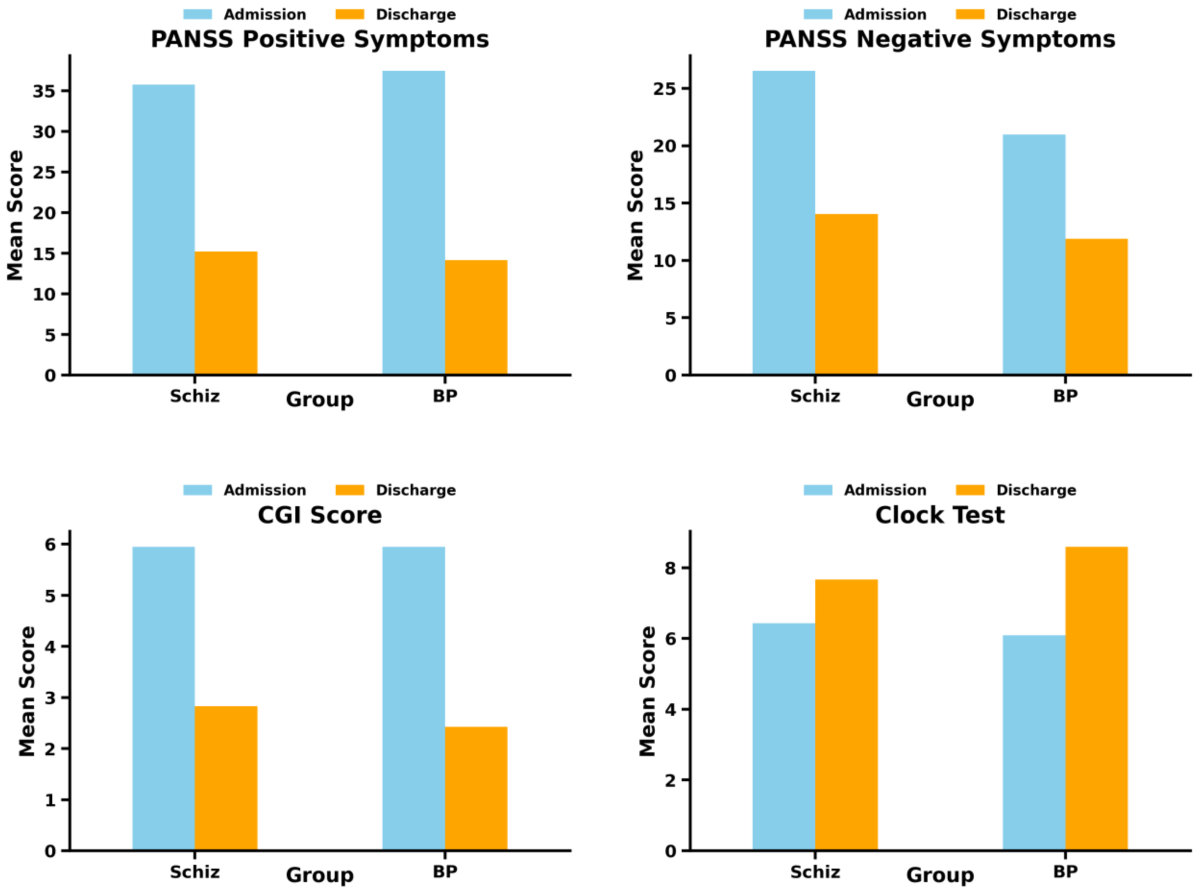
Admission
Discharge
PANSS Positive Symptoms
Mean Score
0
5
10
15
20
25
30
35
Schiz
Group
BP
Admission
Discharge
PANSS Negative Symptoms
Mean Score
0
5
10
15
20
25
Schiz
Group
BP
Admission
Discharge
CGI Score
Mean Score
0
1
2
3
4
5
6
Schiz
Group
BP
Admission
Discharge
Clock Test
Mean Score
0
2
4
6
8
Schiz
Group
BP

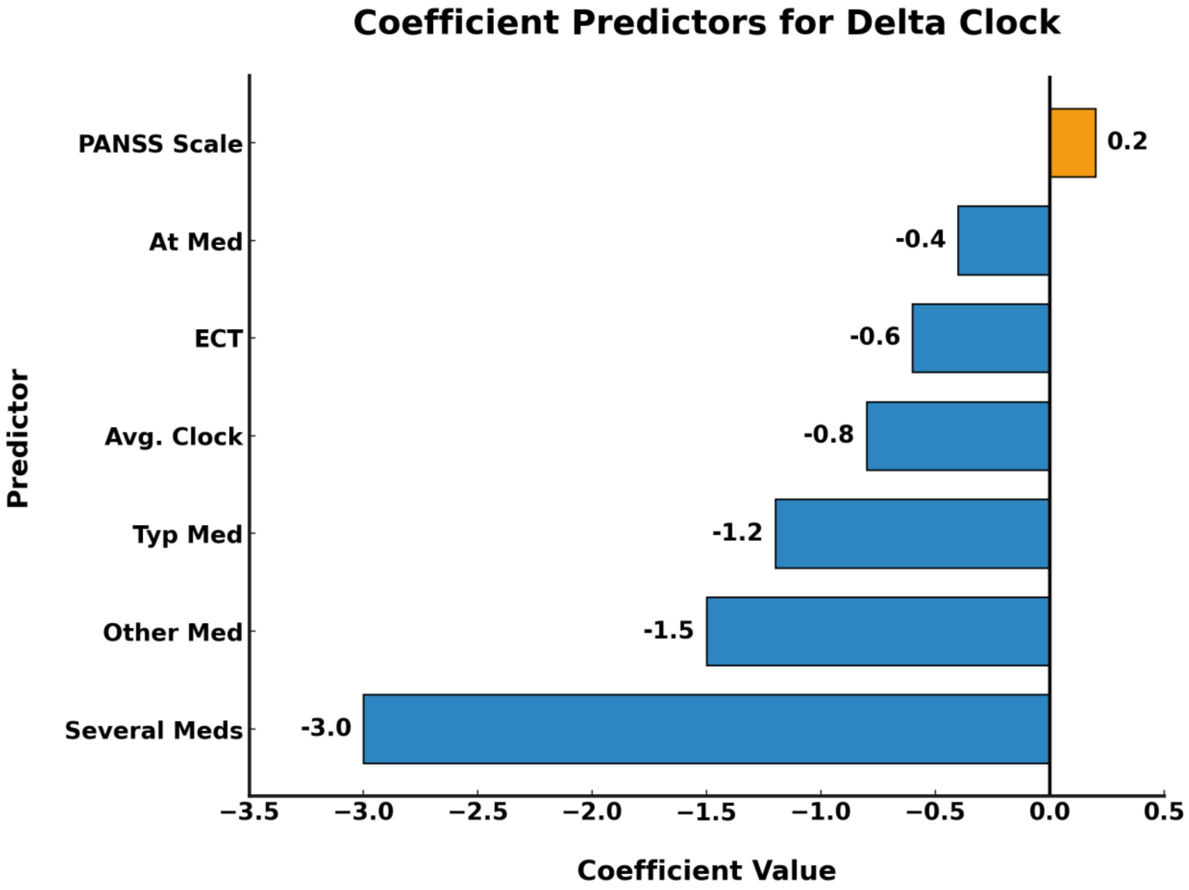

Coefficient Predictors for Delta Clock
Predictor
PANSS Scale
At Med
ECT
Avg. Clock
Typ Med
Other Med
Several Meds
0.2
-0.4
-0.6
-0.8
-1.2
-1.5
-3.0
−3.5
−3.0
−2.5
−2.0
−1.5
−1.0
−0.5
0.0
0.5
Coefficient Value
