# Supplementary material for: Differential cognitive and clinical improvements in Schizophrenia and bipolar disorder following hospitalization: A comparative analysis based on the Clock Drawing Test
Source: PLoS One. 2025 Jul 8;20(7):e0325537. doi: 10.1371/journal.pone.0325537 (PMC12237019; doi:10.1371/journal.pone.0325537)
Supplement: Attachment — Submitted filename: Response to Reviewers R1.docx [file pone.0325537.s005.docx]

***Iran University of Medical Sciences***

**Date**: 02/25/2025

**Revision of manuscript:** *"* *Differential Cognitive and Clinical Improvements in Schizophrenia and Bipolar Disorder Following Hospitalization: A Comparative Analysis Based on the Clock Drawing Test"*

Dear Editor,

We sincerely thank you and the reviewers for your insightful comments on our manuscript. We appreciate the opportunity to revise and improve our work based on the constructive feedback provided. We have carefully considered all the reviewers’ comments and revised the manuscript to address their suggestions. For clarity, we have provided a detailed point-by-point response to each comment below. All changes made to the manuscript have been track-changed, and the revised phrases are also provided below for reference. We hope that the revised manuscript meets your expectations, and we are happy to provide further clarifications or revisions as needed.

Yours sincerely,

**Fatemeh Sadat Mirfazeli**

Fatemeh Sadat Mirfazeli; Assistant Professor of Psychiatry; Mental Health Research Center, Psychosocial Health Research Institute, Department of Psychiatry, School of Medicine, Iran University of Medical Sciences, Tehran, Iran

Email: [mirfazeli.f@iums.ac.ir](mailto:mirfazeli.f@iums.ac.ir)

**Editor Comments**

**This holds an interesting potential to the field and I read with enthusiasm. Nevertheless, there are several concerns from definitions to proper interpretation of the findings. I will list as follows:**

1. **Please double-check grammar (e.g. punctuation and verb tense);**

**Response:** Thank you for your valuable comment. We revised the whole manuscript and double-checked the grammar.

2. **Please double-check refs (e.g. formatting and punctuation);**

**Response:** All references were rechecked.

3. **The use of convenience sampling without justification introduces selection bias and limits generalities. Also, the absence of controls further weakens the findings. It'd be interesting a more representative sampling method or refining the existing approaches. For instance, the sample size is vague, relying on an effect size from a prior study without clear assumptions or a power analysis**. **The explanation of its selection, the effects selection and the CIs are essential. The lack of details on inter-rater reliability introduces risks to data accuracy.**

**Response:** We appreciate these comments. We have provided a more detailed explanation regarding the sample size calculation as follows. The sample size was determined using G*Power 3.1.9.4 software, based on effect size estimates from a previous study which examined cognitive performance using the CDT in schizophrenia and Alzheimer’s disease. In their study, a total sample of 100 participants (32 schizophrenia, 32 Alzheimer’s, 36 controls) revealed significant group differences across various clock conditions (F(2,97) = 26.04 to 32.43, p < 0.001). Based on these findings, the estimated effect size (Cohen’s d) for cognitive group differences was 0.4. Given our study design, where each participant is assessed at two-time points (Admission and Discharge) for multiple measures (CDT, PANSS Positive, PANSS Negative, and CGI), a paired t-test or repeated-measures ANOVA is appropriate. Using G*Power, we calculated that with α = 0.05, power = 0.95, effect size = 0.4, and two repeated measurements, a total sample size of 64 participants is required to detect significant effects. The achieved statistical power (0.953) ensures adequate sensitivity for detecting clinically meaningful changes.

Moreover, to ensure inter-rater reliability, all CDT assessments were initially rated by a trained examiner and subsequently confirmed by an assistant professor of psychiatry with expertise in neurocognitive assessments.

**Overall, both Methods and Results need to be considerably reshaped to ensure a rigorous and transparent study. Also, consider that 1. There is no mention of controlling for medication, comorbidities, or treatment duration, which influence the observed differences, 2. Values are provided without effect sizes or CIs, making the results harder to interpret, 3. There is an inconsistency in how SDs are reported as well as decimals,**

**Response:** We repeated the analysis and added regression to the previous analysis. We then added confidence intervals (CIs) to the previous measures. We reported Mean (SD) for parametric and Median [range] for nonparametric measures and tried to report consistent decimals across the manuscript. All measures were rechecked. Additionally, we introduced and measured the cofactor of treatment in three categories: type of medication (antipsychotics, antidepressants, mood regulators, …), number of medications, and prescription of ECT. We rewrote the results and method sections to provide what was requested by the respected reviewers and editor. Table 2 was refined and Figure 1 has been added to the manuscript.

| **Table 2.** Comparison of cognitive and clinical assessment scores at admission and discharge within and between schizophrenia and bipolar disorder (BDP) groups | | | | | |
| --- | --- | --- | --- | --- | --- |
| **Assessment** | **Schizophrenia** | **BDP** | **P-value*** | **%95 CI** | **Effect size** |
| **Admission CDT** | 5 [2– 10] | 5 [1– 10] | 0.53 | [0.002– 0.28] | Mann-Whitney, two-sample rank-sum test(r): 0.068(small) |
| **Discharge CDT** | 9 [4– 10] | 10 [4– 10] | 0.07 | [0.02– 0.39] | Mann-Whitney, two-sample rank-sum test(r): 0.19(small) |
| **P-value^#^** | 0.02 | 0.0002 |  |  |  |
| **%95 CI** | [0.55– 0.76] | [0.65– 0.81] |  | | |
| **Effect size** | paired two-samples test(r): 0.66(large) | paired two-samples test(r): 0.74(large) |  |  |  |
| **Admission PANSS-positive** | 35.76 (6.38) | 39 [10–46] | 0.14 | [0.01– 0.36] | Mann-Whitney, two-sample rank-sum test(r): 0.16(small) |
| **Discharge PANSS-positive** | 15.19 (4.12) | 14 [7–30] | 0.22 | [0.01– 0.34] | Mann-Whitney, two-sample rank-sum test(r): 0.13(small) |
| **P-value^#^** | <0.0001 | <0.0001 |  |  |  |
| **%95 CI** | [-4.39– -2.89] | [0.87– 0.87] |  | | |
| **Effect size** | Cohen's d: -3.48(large) | paired two-samples test: 0.87(large) |  |  |  |
| **Admission PANSS-negative** | 26.52 (9.36) | 20.98 (6.44) | 0.002 | [-1.15– -0.28] | Cohen's d: -0.69(moderate) |
| **Discharge PANSS-negative** | 13 [7– 33] | 9.5 [6 -31] | 0.008 | [0.06– 0.49] | Mann-Whitney, two-sample rank-sum test(r): 0.29(small) |
| **P-value^#^** | <0.0001 | <0.0001 |  |  |  |
| **%95 CI** | [0.87– 0.87] | [0.85– 0.87] |  | | |
| **Effect size** | paired two-samples test: 0.87(large) | paired two-samples test: 0.86(large) |  |  |  |
| **Admission CGI** | 6 [4– 7] | 6 [5– 7] | 0.80 | [0.001– 0.26] | Mann-Whitney, two-sample rank-sum test(r): 0.03(small) |
| **Discharge CGI** | 3 [2– 6] | 2 [2– 4] | 0.005 | [0.09– 0.5] | Mann-Whitney, two-sample rank-sum test(r): 0.31(moderate) |
| **P-value^#^** | <0.0001 | <0.0001 |  |  |  |
| **%95 CI** | [0.88– 0.92] | [0.89– 0.93] |  | | |
| **Effect size** | paired two-samples test: 0.89(large) | paired two-samples test: 0.91(large) |  |  |  |

Moreover, a multiple linear regression analysis was conducted to examine the association between various clinical, treatment and demographic factors with changes in assessment scores. We reported all models for different dependent variables (changes in CDT score, PANSS-positive, PANSS-negative, and CGI scores).

For the predictive factor of changes in CDT score, the regression model explained approximately 51.1% of the variance in CDT improvement (R² = 0.5107, Adjusted R² = 0.428), and the overall regression model was statistically significant (F(12, 71) = 6.176, p < 0.001). Among the predictors, the initial CDT score was significantly associated with changes in the CDT performance (β = -0.4497, p < 0.001), indicating that patients with lower initial CDT scores showed greater improvements post-hospitalization. The intercept was also statistically significant (β = 10.1003, p = 0.00485), suggesting a baseline effect in CDT changes. However, other clinical variables, including the diagnosis of schizophrenia (β = -0.80174, p = 0.1063), PANSS scores, CGI scores, medication use, and demographic variables (age and gender), did not show statistically significant associations with CDT improvement.

These findings suggest that baseline cognitive impairment, rather than diagnosis or treatment-related factors, is the strongest predictor of post-hospitalization cognitive improvement as measured by the CDT.

**The tables lack clear labelling, making it difficult to interpret results, and there aren’t graphs to visually represent the data. Please consider refining those too;**

**Response:** Thank you for your precious comment. It truly enhances the quality of our work. We accordingly made the changes. We added the following figure and changed the labels.

**Figure 1.** Comparison of cognitive and clinical scores at admission and discharge between bipolar disorder (BDP) and schizophrenia patients. Measures include Clock Drawing Test (CDT), PANSS (Positive and Negative), and Clinical Global Impression (CGI) scores.

**4) Results:**

**- Admission**

**The intriguing difference between symptom burden in patients aligns with clinical understanding. The interesting thing is the less prominence within a group with several differences in meds, especially poly use of meds. These comparable outcomes in test kind indicate the notion that there are impairments regardless of the condition, albeit through potentially different mechanisms. However, although important, both tests won't provide a bigger picture to subtle differences in functional outcomes. Comparable global severity and cognitive impairments in both groups highlight the importance of addressing functional outcomes across groups. To strengthen this section, the authors could consider including merged symptom indices, interaction, any possible influence of PANSS, pos and negative, across the tests, etc;**

**- Discharge**

**- Trends in PANSS negative symptoms and scores hint subtle differences that might warrant investigation with larger sample sizes. As mentioned, understanding the correlations and interactions of the discharge measurements would be really interesting to have a comprehension of the gap or what could have occurred to explain the differences. Examining predictors, correlating changes, and subgroup analyses provide more detailed insights into the findings;**

**- The paired tests should be better explored to explain the main clinical implications of the data. Link specific meds to outcomes (e.g. PANSS scores, CGI changes) to evaluate the efficacy of different treatments in each group - even subtle;**

**- Overall, PANSS differences at admission predict scores at discharge, with negative symptoms showing stronger consistency. This is important, although completely different from changes in CGI, for example, where the pos symptoms were continent;**

**Response**: As stated before, we employed multiple linear regression analysis to examine the association between various clinical, treatment and demographic factors with changes in assessment scores. We reported all models for different dependent variables (changes in CDT score, PANSS-positive, PANSS-negative, and CGI scores).For the predictive factor of changes in CDT score, the regression model explained approximately 51.1% of the variance in CDT improvement (R² = 0.5107, Adjusted R² = 0.428), and the overall regression model was statistically significant (F(12, 71) = 6.176, p < 0.001). Among the predictors, the initial CDT score was significantly associated with changes in the CDT performance (β = -0.4497, p < 0.001), indicating that patients with lower initial CDT scores showed greater improvements post-hospitalization. The intercept was also statistically significant (β = 10.1003, p = 0.00485), suggesting a baseline effect in CDT changes. However, other clinical variables, including the diagnosis of schizophrenia (β = -0.80174, p = 0.1063), PANSS scores, CGI scores, medication use, and demographic variables (age and gender), did not show statistically significant associations with CDT improvement.

For the predictive factor of changes in PANSS-positive symptoms, the regression explained 68.1% of the variance (Adjusted R² = 0.627, p < 0.001), indicating a strong predictive model. The initial PANSS-positive score was the only significant predictor (β = 0.72381, p < 0.001), suggesting that patients with higher initial positive symptom severity had greater symptom reductions.

For the predictive factor of changes in PANSS-negative symptoms, the model explained 64.8% of variance (Adjusted R² = 0.589, p < 0.001), suggesting a strong relationship between baseline negative symptoms and discharge improvement. The initial PANSS-negative score was a significant predictor (β = 0.59116, p < 0.001), showing that patients with more severe negative symptoms at admission tended to have greater reductions.

For the predictive factor of changes in CGI, the model explained 41.7% of the variance (Adjusted R² = 0.319, p < 0.001), making it the weakest among the models. The initial CGI score was the only significant predictor (β = 0.2595, p < 0.001), indicating that patients with more severe initial clinical impressions showed greater improvement.

**5) I calculated and noticed that differences were closely linked to baseline severity. Higher baseline positive symptoms strongly predicted changes in positive symptoms (R² = 0.639, p < 0.001), while cognitive had minimal influence. This was interesting in CGI when baseline negative symptoms were higher (interaction p = 0.019). Patients starting with more severe positive symptoms had improvements, which is counterintuitive. For instance, for delta PANSS the differences were really worth investigating. The meds also influenced the outcomes. While running PCA, I observed that 91.6% of the variance in PANSS items was explained by three components, with PC1 (48.3%) likely reflecting overall symptom severity. PC1 correlated with changes in positive symptoms and global severity. This is aligning with observed data a few years ago, but the lack of logical explanation between PANSS and other variables with the fluctuation in scores are worrying. Please expand and improve the Results and Discussion;**

**Response:** The PCA analysis showed that ten principal components have been generated. The result showed around 90% of the variance was explained by three components.

To determine how much each variable is represented in a given component, the square cosine as a quality of representation (Cos2) was computed using the fviz_cos2 function in R. Here a low value means that the variable is not perfectly represented by that component, and a high value, on the other hand, means a good representation of the variable on that component.

The following Figure showed the square cosine value for each variable with respect to the first three principal components.

This suggests that PANSS-related variables drive the main variance in the dataset, while treatment has almost no representation in the PCA structure.

**6) Overall, without proper refinement it's difficult to assess the relevance of the study;**

**Response:** We would like to gratefully thank you for your precious comments. It truly enhanced the quality of our work and we tried to address every change that you requested most importantly in the method, result, and discussion.

**Reviewer #1:**

**The study, "Differential Cognitive and Clinical Improvements in Schizophrenia and Bipolar Disorder Following Hospitalization: A Comparative Analysis Based on the Clock Drawing Test" by Ahmad Pirani et al., examined changes in cognitive function among hospitalized patients with Schizophrenia and Bipolar Disorder with Psychotic Features (BDP) using the Clock Drawing Test (CDT). This study included 84 patients, equally divided between those with Schizophrenia and those with BDP. Patients were evaluated at hospital admission and discharge using the Positive and Negative Syndrome Scale (PANSS) and the Clinical Global Impression (CGI). The results indicated that patients with Schizophrenia showed very little improvement in cognitive function aftertreatment compared to those with BDP, suggesting a potential inherent cognitive deficit associated with Schizophrenia. However, the study lacks information about the treatment or medications provided, and the small sample size limits the strength of its conclusions. Moreover, the study design was not sufficiently robust to fully address the stated hypothesis. The authors also did not assess other critical cognitive domains, such as attention, memory, reasoning, and executive functions. Overall, while the findings are suggestive, additional research with more comprehensive methodologies and larger samples is needed.**

**Response:** We appreciate the reviewer’s meticulous evaluation of our study; it guided us to improve the quality of our work. In response to the suggestions, we repeated the analysis and added regression to the previous analysis. We introduced and measured the cofactor of treatment in three categories: type of medication (antipsychotics, antidepressants, mood regulators, …), number of medications, and prescription of ECT. We rewrote the method and result section to amend the quality of our work based on the respected editor and reviewers.

The sample size was determined using G*Power 3.1.9.4 software, based on effect size estimates from Bozikas et al. (*https://pubmed.ncbi.nlm.nih.gov/12414073/ )*, which examined cognitive performance using the Clock Drawing Test (CDT) in schizophrenia and Alzheimer’s disease. In their study, a total sample of 100 participants (32 schizophrenia, 32 Alzheimer’s, 36 controls) revealed significant group differences across various clock conditions (F(2,97) = 26.04 to 32.43, p < 0.001). Based on these findings, the estimated effect size (Cohen’s d) for cognitive group differences was 0.4.

Given our study design, where each participant is assessed at two-time points (Admission and Discharge) for multiple measures (CDT, PANSS Positive, PANSS Negative, and CGI), a paired t-test or repeated-measures ANOVA is appropriate. Using G*Power, we calculated that with α = 0.05, power = 0.95, effect size = 0.4, and two repeated measurements, a total sample size of 64 participants is required to detect significant effects. The achieved statistical power (0.953) ensures adequate sensitivity for detecting clinically meaningful changes. The sample size calculation ensures the study has enough statistical power to detect meaningful differences in cognitive and clinical outcomes (CDT, PANSS, CGI) between admission and discharge.

Nevertheless, it is mentioned in the limitation section that larger sample size could show subtle cognitive differences between groups.

“A multiple linear regression analysis was conducted to examine the association between various clinical and demographic factors with cognitive improvements, as measured by changes in the Clock Drawing Test (delta.Clock.score). The model explains approximately 51.1% of the variance in CDT improvement (R² = 0.5107, Adjusted R² = 0.428), and the overall regression model is statistically significant (F(12, 71) = 6.176, p < 0.001).

Among the predictors, the initial Clock Drawing Test score was significantly associated with changes in the CDT performance (β = -0.4497, p < 0.001), indicating that patients with lower initial CDT scores showed greater improvements post-hospitalization. The intercept was also statistically significant (β = 10.1003, p = 0.00485), suggesting a baseline effect in CDT changes. However, other clinical variables, including the diagnosis of schizophrenia (β = -0.80174, p = 0.1063), PANSS scores, CGI scores, medication use, and demographic variables (age and gender), did not show statistically significant associations with CDT improvement.”

-Other cognitive measures could be measured in future studies and are mentioned in the limitation part.

**Reviewer #2:**

**Dear Editor,**

**I appreciate the opportunity to review the manuscript entitled:**

**"Differential Cognitive and Clinical Improvements in Schizophrenia and Bipolar Disorder Following Hospitalization: A Comparative Analysis Based on the Clock Drawing Test"**

**I commend the authors for describing this critical and timely issue. The paper is interesting and well-written; however, I would like to highlight some issues that merit revision:**

**In the manuscript, it is not detectable whether an assessment was made of what might be a protective factor through psychotherapy and counseling intervention, which people often use without prescription to prevent or alleviate symptoms at the onset. I would ask the authors if this was evaluated during the survey and add a short paragraph on this issue; if data are unavailable, add them to the limitations.**

**Response:** We thank the reviewer for the insightful comments. All the patients received occupational therapy through hospitalization whenever they were mentally stable. Regarding the treatment type, we have now included the type of treatment as a cofactor in our revised analyses. All necessary details have been added to the manuscript.
