# Supplementary material for: Differential cognitive and clinical improvements in Schizophrenia and bipolar disorder following hospitalization: A comparative analysis based on the Clock Drawing Test
Source: PLoS One. 2025 Jul 8;20(7):e0325537. doi: 10.1371/journal.pone.0325537 (PMC12237019; doi:10.1371/journal.pone.0325537)
Supplement: Attachment — Submitted filename: PLOS one Review 2025.docx [file pone.0325537.s004.docx]

Thank you for the opportunity to review the manuscript, "Differential Cognitive and Clinical Improvements in Schizophrenia and Bipolar Disorder Following Hospitalization: A Comparative Analysis Based on the Clock Drawing Test," for *PLOS One*.

The purpose of this study was to examine changes in cognitive function and associated positive and negative symptoms among participants with schizophrenia and bipolar disorder with psychotic features (BDP). This study was cross-sectional and included 84 participants who were hospitalized at two psychiatric hospitals; participants were equally divided between both groups. Participants were evaluated at two timepoints (hospital admission and discharge) using the Positive and Negative Syndrome Scale (PANSS), the Clinical Global Impression (CGI), and the Clock Drawing Test (CDT). Within- and between- group changes on the outcome variables were assessed. The results indicated that participants with schizophrenia evidenced less improvement in cognitive function at post-treatment compared to participants with BDP, regardless of change in positive and negative symptoms but related to baseline cognitive profile. Findings suggest underlying cognitive deficits associated with schizophrenia.

I appreciate the focus of this study—as it appears that research examining cognitive functioning in bipolar disorder is limited. The manuscript is written clearly and the preliminary findings from this study have the potential for scientific contributions, which may later inform future research and clinical care. The manuscript may be further strengthened by the following comments and suggestions. As such, I am recommending that the following areas be addressed before the manuscript is considered for publication in *PLOS One*.

General

1. Please check that the manuscript is in accordance to APA style 7^th^ Edition (e.g., spell out all acronyms, report *p* values less than .001 as “*p* < .001,” etc.)
2. Please be mindful of consistency in formatting (e.g., the first letters of “clock drawing test” are capitalized in some places in lowercase in other places, etc.)

Abstract

1. It might be helpful for the abstract to include information about participant demographics (e.g., age, race/ethnicity, gender) and to note the statistical methods employed.
2. Consider tempering language/conclusions since the study is examining associations among outcome variables. This is particularly important because the Clock Drawing Test is typically used as a screener for cognitive impairment in participants with schizophrenia. From this one score, I would use caution in concluding that less pronounced improvements on one cognitive screening measure suggest underlying cognitive deficits for participants with schizophrenia.

Introduction

1. Overall, the introduction is easy to read and well written. I appreciate the general literature review and that the authors acknowledged gaps in the literature and provided rationale for how the study addresses those gaps.
2. One of the aims of this study is to measure cognitive and clinical improvements after treatment. Although briefly addressed in the Discussion, the Introduction does not review relevant treatments and expected treatment response/clinical outcomes for these disorders, especially with regard to the study’s primary outcomes (i.e., positive/negative symptoms, cognition). Inclusion of this background information would provide a better context for the study results and would facilitate a discussion of potential clinical applications of study results in the Discussion.
3. Please clearly state study hypotheses and predictions that accompany study objectives.

Method

1. The timeframe that these data were collected is September 2021 to December 2022. Given that some data were collected during COVID-19, I’m wondering how the historical context may or may not affect study results (e.g., potentially exacerbate disease symptoms).
2. The credentials of the people making the schizophrenia and BDP diagnoses and how the diagnoses were made (e.g., comprehensive evaluation? diagnostic clinical interviewing? by history?) to determine if a participant met diagnostic criteria are not clear.
3. Participants were assessed at two timepoints (admission and discharge); however, it is not clear how many days participants were hospitalized during that timeframe. It would be helpful to include the average time (and SD) from admission to discharge for each group. I’m wondering if duration of hospital stay could be a factor influencing study results.
4. Authors state that all participants received occupational therapy. How often/how much OT did participants receive? I’m wondering if OT intervention duration plays a role in disorder symptoms and cognitive outcomes.
5. It was reported that participants received different biological treatments (i.e., medication and ECT). However, it is unclear how many participants received medication, ECT, or both treatments. What proportion of participants received medication, ECT, or a combination that could alter the frequency/severity of their symptoms and level of cognitive functioning?
6. Of the participants taking medication, how many were on medication for pre- and post-testing? Perhaps medication type positively or negatively affects CDT test performance and scores on the other measures.
7. The authors report demographic data for participant age and gender, but no additional demographic data is provided (i.e., race/ethnicity, primary language, comorbidity, SES, etc.). It is unclear if groups differ on these additional pre-treatment variables; not providing these data notably limit interpretation and generalizability of study results.
8. Relatedly, the demographic data for the total sample is provided in Table 1 but it is not provided for each group. Table 1 could be enhanced by reporting demographic data for both samples. Please see an example below:

| Variable | Schizophrenia  (*n*=42) | | | BDP  (*n*=42) | | | 95% CI | Effect size | *p* |
| --- | --- | --- | --- | --- | --- | --- | --- | --- | --- |
|  | *M* | *SD* | | *M* | *SD* | |  |  |  |
| Gender (male/female) | 51/33 | | | / | | |  | -- |  |
| Age |  |  | |  |  | |  |  |  |
| Race/ethnicity (W/B/H/M) |  | | |  | | |  | -- |  |
| SES |  |  | |  |  | |  |  |  |
| Biological treatment status |  | | |  | | |  |  |  |
| Medication (No/Yes) |  | | |  | | |  | -- |  |
| ECT (No/Yes) |  | | |  | | |  | -- |  |
| Combination (No/Yes) |  | | |  | | |  | -- |  |
| Duration of OT (minutes) |  | |  |  | |  |  |  |  |
| Comorbidity (No/Yes) |  | | |  | | |  | -- |  |
| Duration of hospital stay (days) |  | | |  | | |  |  |  |

9. I appreciate the detailed information about the instruments used in this study. Please also include the range of possible scores for each measure as well as instrument psychometrics (i.e., evidence for reliability, internal and external validity).
10. Were there any outliers? If so, how were those defined and handled?
11. The authors reported comparing the mean changes in each test score (discharge vs. admission) between the two groups. I'm curious if the authors considered using pre-treatment scores as a covariate, especially since schizophrenia participants had significantly higher baseline PANSS-negative scores, to test whether post-treatment means, adjusted for pre-treatment scores, differed between the two groups.
12. This may be beyond the scope of the study, but I'm curious as to what a mixed-model ANOVA (group x symptom domain [positive, negative, cognitive, CDT] x time) would yield with regard to the interaction terms.

Results

1. Table 2 does not clearly show the data in an organized manner, and it is difficult to follow. Consider the following table:

| Variable | Schizophrenia  (*n*=42) | | BDP  (*n*=42) | | 95% CI | Effect size | *p* |
| --- | --- | --- | --- | --- | --- | --- | --- |
|  | *M* | *SD* | *M* | *SD* |  |  |  |
| Pre-treatment/Admission |  | |  | |  |  |  |
| CDT |  |  |  |  |  |  |  |
| PANNS Positive |  |  |  |  |  |  |  |
| PANNS Negative |  |  |  |  |  |  |  |
| Post-treatment/Discharge |  |  |  |  |  |  |  |
| CDT |  |  |  |  |  |  |  |
| PANNS Positive |  |  |  |  |  |  |  |
| PANNS Negative |  |  |  |  |  |  |  |

2. The severity of positive psychotic symptoms was found to be similar across both groups. Could this be a result of sampling bias (positive symptoms are likely more noticeable/impairing than negative symptoms, prompting hospitalization at a psychiatric facility)?

Discussion

1. I appreciate that the authors provide rationale and evidence from literature to explain why there were more pronounced improvements in cognitive function at post-treatment for the BDP group than for the schizophrenia group. However, the authors do not address the potential effects of biological treatment and intervention (i.e., OT) on these outcomes. Perhaps the authors could comment on treatment/intervention factors that might influence primary outcomes, using previous literature on expected treatment response/clinical outcomes for these disorders in the explanation.
2. I appreciate that the authors provide an implication for clinical practice. However, the implication seems relatively general and to lack clear, detailed guidelines for actionable steps, which limits the applicability of findings for researchers and practitioners. It would be helpful to expand this implication and to provide guidance for researchers and practitioners to follow. For example, earlier in the manuscript the authors mention cognitive intervention as a potential effective treatment for these disorders, but it was not further addressed in the Discussion as a potential future direction for the field.
3. In terms of the limitations, I appreciate that the authors acknowledge that the study’s relatively small sample size is a limitation. However, the authors do not address that the majority of study participants identify as male, which also limits generalizability. I find myself looking to the context to help explain why the sample mostly identifies as male, but little context is provided.
4. Perhaps authors could comment on potential sampling bias and limits to generalizability with regard to collecting data during COVID-19 and to collecting data from study participants who were hospitalized. Another potential limitation may be that the study lacked a control group.
