# Supplementary material for: Differential cognitive and clinical improvements in Schizophrenia and bipolar disorder following hospitalization: A comparative analysis based on the Clock Drawing Test
Source: PLoS One. 2025 Jul 8;20(7):e0325537. doi: 10.1371/journal.pone.0325537 (PMC12237019; doi:10.1371/journal.pone.0325537)
Supplement: Attachment — Submitted filename: plos one-response R2.docx [file pone.0325537.s006.docx]

We sincerely thank you and the reviewers for your valuable time and insightful comments on our manuscript. We appreciate the opportunity to revise and improve our work based on the constructive feedback we have received. We have carefully considered all the reviewers’ comments and have made the necessary revisions to the manuscript to address their suggestions. For clarity, we have provided a detailed point-by-point response to each comment below. Reviewer comments are presented in **bold**, followed by our responses in regular text. All changes made to the manuscript have been highlighted, and the revised phrases are also provided below for reference. We hope that the revised manuscript meets your expectations, and we are happy to provide further clarifications or revisions as needed.

Thank you for considering our revised manuscript for publication in PLOS ONE.

**Comments to the Author**

6. Review Comments to the Author

Please use the space provided to explain your answers to the questions above. You may also include additional comments for the author, including concerns about dual publication, research ethics, or publication ethics. (Please upload your review as an attachment if it exceeds 20,000 characters)

**Reviewer #1: (No Response)**

**Reviewer #2: The paper is very interesting and well-written, methodologically unexceptionable, and the new implementations provide a valid contribution to the work. Every requested correction has been done, and the manuscript is now suitable for publication.**

**Reviewer #3: It is also uploaded and attached.**

**Thank you for the opportunity to review the manuscript, "Differential Cognitive and Clinical Improvements in Schizophrenia and Bipolar Disorder Following Hospitalization: A Comparative Analysis Based on the Clock Drawing Test," for PLOS One.

The purpose of this study was to examine changes in cognitive function and associated positive and negative symptoms among participants with schizophrenia and bipolar disorder with psychotic features (BDP). This study was cross-sectional and included 84 participants who were hospitalized at two psychiatric hospitals; participants were equally divided between both groups. Participants were evaluated at two timepoints (hospital admission and discharge) using the Positive and Negative Syndrome Scale (PANSS), the Clinical Global Impression (CGI), and the Clock Drawing Test (CDT). Within- and between- group changes on the outcome variables were assessed. The results indicated that participants with schizophrenia evidenced less improvement in cognitive function at post-treatment compared to participants with BDP, regardless of change in positive and negative symptoms but related to baseline cognitive profile. Findings suggest underlying cognitive deficits associated with schizophrenia.**

**I appreciate the focus of this study—as it appears that research examining cognitive functioning in bipolar disorder is limited. The manuscript is written clearly and the preliminary findings from this study have the potential for scientific contributions, which may later inform future research and clinical care. The manuscript may be further strengthened by the following comments and suggestions. As such, I am recommending that the following areas be addressed before the manuscript is considered for publication in PLOS One.**

**General
1. Please check that the manuscript is in accordance to APA style 7th Edition (e.g., spell out all acronyms, report p values less than .001 as “p < .001,” etc.)**

Thank the reviewer for the precious feedback, we did that. We organized manuscript based on APA style 7th Edition and check other requests based on your useful comment.

2. Please be mindful of consistency in formatting (e.g., the first letters of “clock drawing test” are capitalized in some places in lowercase in other places, etc.)
Thank the reviewer for your precious feedback, we corrected all.

Abstract

**1. It might be helpful for the abstract to include information about participant demographics (e.g., age, race/ethnicity, gender) and to note the statistical methods employed.**

Thank the reviewer, we changed that as well:

“This cross-sectional study enrolled 84 patients (42 schizophrenia, 42 BDP, 51 male and 33 female) aged 42.85 (±11.51), ranging from 20 to 65 years old, from two psychiatric hospitals in Iran”

“Within-group and between-group changes were analyzed using **paired t-tests** for pre-post comparisons and multiple regression was used to assess predictive factors of changes in cognitive and symptom changes.”

**2. Consider tempering language/conclusions since the study is examining associations among outcome variables. This is particularly important because the Clock Drawing Test is typically used as a screener for cognitive impairment in participants with schizophrenia. From this one score, I would use caution in concluding that less pronounced improvements on one cognitive screening measure suggest underlying cognitive deficits for participants with schizophrenia.**

Thank the reviewer for pointing out this important matter.

“While both groups exhibited cognitive and clinical improvements following hospitalization, patients with schizophrenia showed relatively less improvement in the CDT compared to those with BDP. These findings may suggest that cognitive recovery may follow a different trajectory in schizophrenia, independent of changes in positive and negative symptoms but related to the initial cognitive profile. However, given that the CDT is a screening tool rather than a comprehensive cognitive assessment, these results should be interpreted with caution. Future research should incorporate broader neurocognitive assessments to better understand the cognitive trajectories of these populations “

**Introduction
1. Overall, the introduction is easy to read and well written. I appreciate the general literature review and that the authors acknowledged gaps in the literature and provided rationale for how the study addresses those gaps**.

We thank the reviewer for this comment

**2. One of the aims of this study is to measure cognitive and clinical improvements after treatment. Although briefly addressed in the Discussion, the Introduction does not review relevant treatments and expected treatment response/clinical outcomes for these disorders, especially with regard to the study’s primary outcomes (i.e., positive/negative symptoms, cognition). Inclusion of this background information would provide a better context for the study results and would facilitate a discussion of potential clinical applications of study results in the Discussion.**

Thank the reviewer for the valuable comments to make our paper better.

„Treatment approaches for SCZ and BDP typically involve a combination of pharmacotherapy, psychotherapy, and, in some cases, somatic interventions like electroconvulsive therapy (ECT), aimed at reducing symptom severity and improving functional outcomes. For SCZ, antipsychotic medications are the cornerstone of treatment, effectively reducing positive symptoms such as hallucinations and delusions within weeks, though negative symptoms and cognitive deficits often show limited improvement (13). A meta-analysis by Leucht et al. (2017) found that antipsychotics yield moderate effect sizes (Cohen’s d ≈ 0.5) for positive symptom reduction, but cognitive gains are less consistent, with some studies reporting minor improvements in processing speed and attention following second-generation antipsychotics (14). In BDP, mood stabilizers (e.g., lithium, valproate) combined with antipsychotics during psychotic manic episodes are standard, with evidence suggesting robust reductions in positive symptoms and mania-related cognitive disruptions within 4-6 weeks. A review by Nierenberg et al. (2023) highlights that bipolar patients often experience partial cognitive recovery post-treatment, particularly in executive functioning, though deficits may persist during euthymic phases (5). These differential treatment responses underscore the need to evaluate how clinical and cognitive outcomes align in hospitalized patients, providing a foundation for understanding potential therapeutic impacts in this study’s context.“

**3. Please clearly state study hypotheses and predictions that accompany study objectives.**

Done:

“We hypothesized that: (1) both schizophrenia and BDP patients would exhibit significant improvements in cognitive function (measured by CDT) and clinical symptoms (measured by PANSS and CGI) following hospitalization, given the expected efficacy of standard treatments; (2) BDP patients would demonstrate greater improvements in CDT scores and positive symptom reduction compared to schizophrenia patients, reflecting evidence of less severe baseline cognitive deficits and stronger treatment response in bipolar disorder; and (3) changes in cognitive function would not strongly correlate with changes in positive or negative symptoms in either group, suggesting that cognitive deficits may represent a distinct domain from psychotic symptomatology. “

These predictions were based on prior findings of differential cognitive impairment and treatment responsiveness between the two disorders (5, 10, 23)

**Method
1. The timeframe that these data were collected is September 2021 to December 2022. Given that some data were collected during COVID-19, I’m wondering how the historical context may or may not affect study results (e.g., potentially exacerbate disease symptoms).**

We thank the reviewer for highlighting the important historical context of the COVID-19 pandemic during our data collection period (September 2021 to December 2022) and its potential influence on study outcomes. To mitigate the direct impact of COVID-19 on our sample, we included only patients who tested negative for COVID-19 at admission, as confirmed by hospital protocols at Rasoul Akram Hospital and Iran Psychiatry Hospital. This exclusion criterion ensured that our findings were not confounded by the acute effects of the virus on symptom presentation or cognitive function in schizophrenia and BDP patients.

However, we acknowledge that the broader context of the pandemic may have indirectly influenced our results. During this period, public health measures, fear of infection, and strained healthcare systems likely affected patients’ access to routine psychiatric care, potentially leading to delayed treatment-seeking and more severe symptom presentations at admission. This aligns with literature suggesting that the COVID-19 pandemic exacerbated mental health outcomes, with studies reporting increased relapse rates and symptom severity in schizophrenia and bipolar disorder due to reduced outpatient care and heightened psychosocial stress . In our study, we observed relatively high baseline PANSS scores (e.g., mean PANSS-positive: 35.76 in schizophrenia, 39 in BDP) and low CDT scores (median: 5 in both groups) at admission, which may reflect such delays in care, though we did not directly assess this hypothesis. Additionally, hospital conditions during the pandemic—such as reduced staffing, visitor restrictions, or altered treatment protocols—may have impacted the quality and consistency of inpatient care, potentially influencing clinical and cognitive improvements.

As evaluating the indirect effects of the COVID-19 pandemic was not an objective of our study, we did not collect data on variables such as time since last outpatient visit, changes in relapse frequency, or hospital resource constraints. This represents a limitation, as these factors could have contributed to the observed outcomes, particularly the severity of symptoms at admission and the extent of recovery by discharge. We have added this consideration to the revised Discussion under limitations, noting that future studies should explore the pandemic’s indirect effects on psychiatric populations by incorporating variables such as treatment access, relapse history, and hospital conditions during crisis periods. Such research could provide valuable insights into how systemic factors influence recovery trajectories in schizophrenia and BDP.

2**. The credentials of the people making the schizophrenia and BDP diagnoses and how the diagnoses were made (e.g., comprehensive evaluation? diagnostic clinical interviewing? by history?) to determine if a participant met diagnostic criteria are not clear.**

**Thank the reviewer for the valuable comment**

“Inclusion criteria were an age of 18 and above, a diagnosis of schizophrenia or BDP according to the Diagnostic and Statistical Manual of Mental Disorders 5 (DSM-5), and consent to participate in the study. The diagnosis was made by a psychiatry attending using a comprehensive psychiatric interview. “

It is added.

3. **Participants were assessed at two timepoints (admission and discharge); however, it is not clear how many days participants were hospitalized during that timeframe. It would be helpful to include the average time (and SD) from admission to discharge for each group. I’m wondering if duration of hospital stay could be a factor influencing study results.**

Unfortunately, we did not record the duration of the hospital of the patient back then and we could find only 25 patients with their names because we need a national code to access all patients' records. The average stay of those 25 patients is 21.28 days. It is usually the average stay of patients in hospitals in Iran. Patients are usually hospitalized from 14 to 28 days. Given the lack of comprehensive duration data, we could not assess its impact on cognitive (CDT) or clinical (PANSS, CGI) outcomes in full. This represents a limitation of the study, as longer hospital stays could theoretically enhance treatment exposure and influence symptom or cognitive recovery. We have noted this in the revised Discussion under limitations and recommend that future studies systematically record and analyze hospitalization duration as a potential covariate.

**4. Authors state that all participants received occupational therapy. How often/how much OT did participants receive? I’m wondering if OT intervention duration plays a role in disorder symptoms and cognitive outcomes.**

All participants received occupational therapy (OT) twice weekly throughout their hospitalization, with each session lasting approximately 45 minutes, consistent with standard inpatient psychiatric care protocols at the study sites (Rasoul Akram Hospital and Iran Psychiatry Hospital). As OT was uniformly administered to all patients in both the schizophrenia and BDP groups, it was not included as a variable in the multiple regression analyses. This decision was based on the lack of variability in OT exposure across participants, which precluded its evaluation as a predictor of differential changes in cognitive (CDT) or clinical (PANSS, CGI) outcomes. While OT duration or frequency could theoretically influence symptom severity and cognitive function, the consistent application in this study design limits our ability to assess its specific contribution. Future studies with variable OT dosing could explore its potential moderating effects on these outcomes.

**5. It was reported that participants received different biological treatments (i.e., medication and ECT). However, it is unclear how many participants received medication, ECT, or both treatments. What proportion of participants received medication, ECT, or a combination that could alter the frequency/severity of their symptoms and level of cognitive functioning?**

We updated the Table 1 for demographic characteristics of the participants

Table 1. Demographic characteristics of the participants

| Variable | | Mean$\pm$SD/Median[min-max]/ No.[%] | | 95% CI | Effect size | p |
| --- | --- | --- | --- | --- | --- | --- |
|  |  | Groups(n=84) | |  |  |  |
|  |  | Schizophrenia | BDP |  |  |  |
| Age(y) | | 39[24-65] | 40.74$\pm$10.82 | [0.0035-0.25] | 0.02(small) | P=0.85 |
| Number of participants | | 42[%50] | 42[%50] | - | - | - |
| Gender | Female: 33[%39.3] | 15[%35] | 18[%42] | - | Cohen's w: 0.26(moderate) | p < 0.01 |
|  | Male: 51[%60.7] | 27[%65] | 24[%58] |  |  |  |
| ECT | | 10[%24] | 6[%14] | - | Cohen's w: 0.09 (small) | P=0.26 |
| Number of Medication | | 2[1-4] | 2[1-3] | [0.13- 0.53] | 0.34(moderate) | p < 0.01 |
| Mood stabilizer | | 10[%24] | 38[%90] | - | Cohen's w: 0.64(large) | 6.7e-10 |
| Antipsychotic medication | | 42[%100] | 40[%95] | - | - | - |

**6. Of the participants taking medication, how many were on medication for pre- and post-testing? Perhaps medication type positively or negatively affects CDT test performance and scores on the other measures.**

"Of the participants taking medication, how many were on medication for pre- and post-testing? Perhaps medication type positively or negatively affects CDT test performance and scores on the other measures."

Revised Response:
"We appreciate the reviewer’s insightful question regarding the potential influence of medication on CDT performance and other clinical measures (PANSS, CGI). In our study, medication history was recorded on the first day of hospitalization based on patient self-reports and available medical records. However, we acknowledge that self-reports in this context may be unreliable, as patients experiencing acute relapse—particularly those with schizophrenia or BDP—frequently underreport or overreport medication adherence due to symptom severity, lack of insight, or stigma. For this reason, and because pre-admission medication adherence could not be verified objectively, we did not include pre-admission medication status as a variable in our regression analyses.

During hospitalization, all participants received standardized pharmacological treatment as per hospital protocols, which included antipsychotics for schizophrenia patients and a combination of mood stabilizers and antipsychotics for BDP patients, alongside twice-weekly occupational therapy. On the day of admission, some participants received one or two PRN (as-needed) doses of antipsychotics or benzodiazepines to manage acute agitation before the initial assessments (CDT, PANSS, CGI). By the time of discharge assessments, all participants had been on their prescribed inpatient medication regimens for the duration of their hospital stay (mean = 21.28 days, based on a subset of 25 patients). While medication type and adherence could theoretically influence cognitive and clinical outcomes—particularly given evidence that second-generation antipsychotics may improve cognitive function in schizophrenia —the lack of reliable pre-admission data and the variability in PRN use on admission day precluded a detailed analysis of medication effects in this study.

This represents a limitation, as differences in medication status or type (e.g., antipsychotics vs. mood stabilizers) may have contributed to the observed improvements in CDT scores (e.g., greater gains in BDP patients) or symptom reduction (e.g., PANSS-positive reductions). We have added this limitation to the revised discussion, noting that future studies should incorporate objective measures of medication adherence (e.g., pharmacy records, blood levels) and detailed medication histories (e.g., type, dosage, duration) to better assess their impact on cognitive and clinical outcomes in schizophrenia and BDP."

**7. The authors report demographic data for participant age and gender, but no additional demographic data is provided (i.e., race/ethnicity, primary language, comorbidity, SES, etc.). It is unclear if groups differ on these additional pre-treatment variables; not providing these data notably limit interpretation and generalizability of study results.**

Thank the reviewer for the valuable comment. All the patients were Iranian, and Farsi was their primary language. They did not have any neurological comorbidity. However, their socioeconomic status was not recorded.

“Inclusion criteria were an age of 18 and above, Farsi speaking and Iranian population, a diagnosis of schizophrenia or BDP according to the Diagnostic and Statistical Manual of Mental Disorders 5 (DSM-5), and consent to participate in the study. The diagnosis was made by a psychiatrist attending using a comprehensive psychiatric interview. The exclusion criteria included a history of traumatic brain injury, seizure within the last two years, brain tumor, and central nervous system (CNS) diseases such as multiple sclerosis.”

**8. Relatedly, the demographic data for the total sample is provided in Table 1 but it is not provided for each group. Table 1 could be enhanced by reporting demographic data for both samples. Please see an example below:**

Variable Schizophrenia
(n=42) BDP
(n=42) 95% CI Effect size p
M SD M SD
Gender (male/female) 51/33 / --
Age
Race/ethnicity (W/B/H/M) --
SES
Biological treatment status
Medication (No/Yes) --
ECT (No/Yes) --
Combination (No/Yes) --
Duration of OT (minutes)
Comorbidity (No/Yes) --
Duration of hospital stay (days)

Table 1 is updated as requested. All patients received medication therefore we did not add a cell showing a combination of ECT and medication. All the patients were Iranian, and Farsi was their primary language.

| Variable | | Mean$\pm$SD/Median[min-max]/ No.[%] | | 95% CI | Effect size | p |
| --- | --- | --- | --- | --- | --- | --- |
|  |  | Groups(n=84) | |  |  |  |
|  |  | Schizophrenia | BDP |  |  |  |
| Age(y) | | 39[24-65] | 40.74$\pm$10.82 | [0.0035-0.25] | 0.02(small) | P=0.85 |
| Number of participants | | 42[%50] | 42[%50] | - | - | - |
| Gender | Female: 33[%39.3] | 15[%35] | 18[%42] | - | Cohen's w: 0.26(moderate) | p < 0.05 |
|  | Male: 51[%60.7] | 27[%65] | 24[%58] |  |  |  |
| ECT | | 10[%24] | 6[%14] | - | Cohen's w: 0.09 (small) | P=0.26 |
| Number of Medication | | 2[1-4] | 2[1-3] | [0.13- 0.53] | 0.34(moderate) | p < 0.01 |
| Mood stabilizer | | 10[%24] | 38[%90] | - | Cohen's w: 0.64(large) | 6.7e-10 |
| Antipsychotic medication | | 42[%100] | 40[%95] | - | - | - |

**9. I appreciate the detailed information about the instruments used in this study. Please also include the range of possible scores for each measure as well as instrument psychometrics (i.e., evidence for reliability, internal and external validity).**

*Instruments*

**a. Positive and Negative Scale Symptoms (PANSS)**

The PANSS, developed by Kay, Opler, and Fiszbein in 1987, is a widely used psychometric tool for assessing the severity of psychosis symptoms in schizophrenia and bipolar disorder with psychotic features (BDP) (4, 16). It consists of 30 items divided into three subscales: positive (7 items), negative (7 items), and general psychopathology (16 items). In this study, we focused on the positive (P-PANSS) and negative (N-PANSS) subscales. Each item is scored on a 7-point scale (1 = absent, 7 = extreme), yielding a total score range of 7 to 49 for each subscale, with higher scores indicating greater symptom severity. The PANSS has demonstrated strong psychometric properties, with high internal consistency (Cronbach’s α = 0.73–0.83 for subscales) and test-retest reliability (r = 0.77–0.89) in schizophrenia populations (16). Its construct validity is supported by factor analyses confirming the three-subscale structure, and external validity is evidenced by correlations with other clinical measures like the Brief Psychiatric Rating Scale (BPRS) (r = 0.64–0.81) (16, 39).

**b. Clinical Global Impression (CGI)**

The CGI is a clinician-rated tool used to assess the severity and change in a patient’s condition, commonly applied in psychiatric research and clinical practice (17). In this study, we used the CGI-Severity (CGI-S) subscale, which rates illness severity on a 7-point scale (1 = normal, not at all ill; 7 = among the most extremely ill), with a score range of 1 to 7; higher scores indicate greater severity. The CGI has shown adequate reliability, with inter-rater reliability coefficients ranging from 0.71 to 0.8 in psychiatric populations (17, reff). Its external validity is evidenced by moderate correlations with symptom-specific scales like the PANSS (r = 0.36 to 0.61) (refff). However, the CGI’s subjective nature can introduce variability, which we mitigated by using trained psychiatric specialists for assessments.

**c. Clock Drawing Test (CDT)**

The CDT has been widely used as a measure of cognitive function in several disorders since the 1960s. CDT has been linked to dysfunction in the parietal lobe, as well as various brain regions such as the left and right posterior and middle temporal lobe, the right middle frontal gyrus, and the right occipital lobes. In this research, participants were provided with a white A4 paper and instructed to draw a clock, setting the time to 2:45. Each clock was quantitatively scored, with the scoring checked by a neuropsychiatrist. We utilized the scoring procedure advocated by Mendes-Santos et al., in which the scores range from 1 to 10 with higher scores indicating better function. (18). The scoring was rated by a trained examiner and rechecked by an assistant professor of psychiatry.

The CDT exhibits good psychometric properties, with sensitivity ranging from 86% to 97% and specificity ranging from 65% to 85% in neurocognitive assessments. Its construct validity is supported by its sensitivity to executive dysfunction and visuospatial deficits, (23, 24) and external validity is evidenced by correlations with comprehensive cognitive batteries like the Mini-Mental State Examination (MMSE) (24) . In SCZ, the CDT has been validated as a screening tool for cognitive impairment, though its specificity may be lower in psychiatric versus organic disorders (25) .

**10. Were there any outliers? If so, how were those defined and handled?**

Thank the reviewer for raising such an important question. No, we did not face any outliers. **11. The authors reported comparing the mean changes in each test score (discharge vs. admission) between the two groups. I'm curious if the authors considered using pre-treatment scores as a covariate, especially since schizophrenia participants had significantly higher baseline PANSS-negative scores, to test whether post-treatment means, adjusted for pre-treatment scores, differed between the two groups.**

**We Thank the reviewer for this delicate matter.**

**“**Since schizophrenia patients had significantly higher baseline PANSS-negative scores, we conducted an ANCOVA to compare post-treatment PANSS-negative scores between the two groups while controlling for baseline levels. The analysis revealed that even after adjustment, the groups differed significantly at discharge (F(1,81) = 5.10, *p* = 0.0266). Baseline PANSS-negative scores also had a strong effect on discharge outcomes (F(1,81) = 42.61, *p* < 0.001). The adjusted post-treatment means were similar between groups: 13.0 for both schizophrenia and BDP, with overlapping confidence intervals, indicating comparable symptom severity after treatment when baseline differences were accounted for.”

**12. This may be beyond the scope of the study, but I'm curious as to what a mixed-model ANOVA (group x symptom domain [positive, negative, cognitive, CDT] x time) would yield with regard to the interaction terms.**

To explore the interaction between time, symptom domain, and diagnosis, we conducted a mixed-model ANOVA with fixed effects for **patient type (schizophrenia vs. BDP)**, **symptom domain (CDT, PANSS-positive, PANSS-negative, CGI)**, and **time (admission vs. discharge)**, including **random intercepts for subjects**.

The analysis revealed significant main effects of **patient type** (*F*(1,82) = 4.08, *p* = 0.0468), **symptom domain** (*F*(3,574) = 841.15, *p* < 2.2e-16), and **time** (*F*(1,574) = 624.07, *p* < 2.2e-16), indicating that overall scores differed between schizophrenia and BDP patients, across clinical and cognitive domains, and between admission and discharge.

There were also significant interactions between:

- **Patient type and symptom domain** (*F*(3,574) = 8.68, *p* < 0.001), suggesting that the diagnostic group differences varied by domain;
- **Symptom domain and time** (*F*(3,574) = 228.01, *p* < 2.2e-16), indicating that changes over time were domain-specific; and
- **The three-way interaction** of patient type × symptom domain × time (*F*(3,574) = 3.56, *p* = 0.0142), reflecting that the pattern of change from admission to discharge across domains differed between the two diagnostic groups.

The **interaction between patient type and time** alone was not significant (*p* = 0.577), implying that, on average, both groups improved similarly over time, but the **nature of that improvement depended on the symptom domain**.

However, we did not add to the manuscript, since it is beyond the scope of our study.

**Results
1. Table 2 does not clearly show the data in an organized manner, and it is difficult to follow. Consider the following table:**

Variable Schizophrenia
(n=42) BDP
(n=42) 95% CI Effect size p
M SD M SD
Pre-treatment/Admission
CDT
PANNS Positive
PANNS Negative
Post-treatment/Discharge
CDT
PANNS Positive
PANNS Negative

The reviewer is right. The table is not clear enough. We changed it.

**Table 2: Comparison of Cognitive and Clinical Measures in Schizophrenia and Bipolar Disorder (BDP) Patients**

| **Assessment** | **Schizophrenia Mean (SD) or Median [Range]** | **BDP Mean (SD) or Median [Range]** | **P-value** | **95% CI** | **Effect Size** |
| --- | --- | --- | --- | --- | --- |
| **Clock Drawing Test (CDT)** | | | | | |
| Admission CDT | 5 [2–10] | 5 [1–10] | P=0.53 | [0.002–0.28] | 0.068 (small) |
| Discharge CDT | 9 [4–10] | 10 [4–10] | P=0.07 | [0.02–0.39] | 0.19(small) |
| **P-value (Admission vs. Discharge)** | - | - | p < 0.05 (SZ)  p < 0.001 (BDP) | [0.55–0.76] (SZ)  [0.65–0.81] (BDP) | **0.66 (Large)** (SZ)  **0.74 (large)** (BDP) |
| **PANSS-Positive** | | | | | |
| Admission PANSS-Positive | 35.76 (6.38) | 39 [10–46] | P=0.14 | [0.01–0.36] | **0.16 (Small)** |
| Discharge PANSS-Positive | 15.19 (4.12) | 14 [0–30] | P=0.22 | [0.01–0.36] | **0.13 (Small)** |
| **(Admission vs. Discharge)** | - | - | **p<0.0001 (SZ)**  **p<0.0001 (BDP)** | [-4.39– -2.89] (SZ)  [0.78–0.87] (BDP) | **3.48 (Large)** (SZ)  **0.87 (Large)** (BDP) |
| **PANSS-Negative** | | | | | |
| Admission PANSS-Negative | 26.52 (9.36) | 20.98 (6.44) | p < 0.01 | [-1.15 – -0.28] | **-0.69 (Large)** |
| Discharge PANSS-Negative | 13 [7–33] | 9.56 [6–31] | p < 0.01 | [0.06–0.49] | **0.29 (Small)** |
| **P-value (Admission vs. Discharge)** | - | - | **p<0.0001 (SZ)**  **p<0.0001 (BDP)** | [0.87–0.87] (SZ) [0.85–0.87] (BDP) | **0.87 (Large)** (SZ) **0.69 (Moderate)** (BDP) |
| **Clinical Global Impression (CGI)** | | | | | |
| Admission CGI | 6 [1–7] | 6 [1–7] | P=0.8 | [0.001–0.26] | **0.03 (Small)** |
| Discharge CGI | 3 [2–6] | 2 [2–4] | p < 0.01 | [0.09–0.5] | **0.31 (Moderate)** |
| **P-value (Admission vs. Discharge)** | - | - | **p<0.0001 (SZ)**  **p<0.0001 (BDP)** | [0.88–0.92] (SZ) [0.89–0.93] (BDP) | **0.89 (Large)** (SZ)  **0.91 (Large)** (BDP) |

* P-value corresponds to between-group comparisons. # P-value corresponds to the admission vs. discharge comparison within each patient group. Data are presented as mean (SD) and median [range]

**2. The severity of positive psychotic symptoms was found to be similar across both groups. Could this be a result of sampling bias (positive symptoms are likely more noticeable/impairing than negative symptoms, prompting hospitalization at a psychiatric facility)?**

We thank the reviewer for raising the important question of whether the observed similarity in positive psychotic symptom severity between schizophrenia and BDP groups (mean PANSS-positive: 35.76 [SD = 6.38] for schizophrenia, 39 [range: 10–46] for BDP) could reflect sampling bias, given that positive symptoms are often more noticeable and impairing, potentially prompting hospitalization. We acknowledge that positive symptoms such as hallucinations and delusions are typically more overt and may lead to earlier recognition and admission in psychiatric settings. However, in our study, clinical records and admission criteria indicated that a significant proportion of hospitalizations were driven by severe negative symptoms as well. Specifically, impaired self-care (e.g., neglecting personal hygiene), prolonged social isolation (e.g., staying at home for extended periods), and inadequate nutritional intake were frequently cited as primary reasons for admission in both groups, often due to their impact on functional impairment and caregiver burden.

Discussion

**1. I appreciate that the authors provide rationale and evidence from literature to explain why there were more pronounced improvements in cognitive function at post-treatment for the BDP group than for the schizophrenia group. However, the authors do not address the potential effects of biological treatment and intervention (i.e., OT) on these outcomes. Perhaps the authors could comment on treatment/intervention factors that might influence primary outcomes, using previous literature on expected treatment response/clinical outcomes for these disorders in the explanation.**

We thank the reviewer for this important point.

“The differential cognitive and clinical improvements observed may also be influenced by the biological treatments and interventions provided during hospitalization, which varied between groups. All participants received OT twice weekly (45-minute sessions), aimed at improving functional skills, while biological treatments differed: SCZ patients were primarily treated with antipsychotics, and BDP patients received a combination of mood stabilizers (e.g., lithium, valproate) and antipsychotics, with some of both groups receiving ECT for severe symptoms. Literature suggests that second-generation antipsychotics, commonly used in SCZ, can yield modest cognitive improvements, but these gains are often limited by the disorder’s underlying neurobiology (33, 34) . In contrast, mood stabilizers and antipsychotics in BDP may facilitate greater cognitive recovery, particularly during manic episodes, by stabilizing mood and reducing psychotic symptoms, with studies reporting moderate effect sizes (≈ 0.4–0.5) on executive function and processing speed post-treatment. ECT, used in some BDP patients, has been associated with rapid symptom reduction but can cause transient cognitive side effects, though these typically resolve within weeks (35) . OT, which focuses on daily living skills and social engagement, may have contributed to improvements in negative symptoms by enhancing functional capacity, with prior research indicating small to moderate effects on social cognition in both disorders (36). However, the uniform application of OT and the lack of detailed medication data (e.g., dosages) in our study limit our ability to fully disentangle their contributions to the observed outcomes, particularly the greater CDT gains in BDP. Future studies should examine the specific effects of treatment type and intensity on cognitive and clinical trajectories in these populations.“

**2. I appreciate that the authors provide an implication for clinical practice. However, the implication seems relatively general and to lack clear, detailed guidelines for actionable steps, which limits the applicability of findings for researchers and practitioners. It would be helpful to expand this implication and to provide guidance for researchers and practitioners to follow. For example, earlier in the manuscript the authors mention cognitive intervention as a potential effective treatment for these disorders, but it was not further addressed in the Discussion as a potential future direction for the field.**

It is added in the manuscript.

”Overall, our research did not uncover any significant relationships between changes in CDT scores and changes in other scales, suggesting that cognitive deficits could act as findings irrespective of positive and negative symptoms. We recommend that clinicians integrate brief, validated cognitive screening tools, such as the Clock Drawing Test (CDT) used in our study or the Montreal Cognitive Assessment (MoCA), into standard assessments at admission, during hospitalization, and at follow-up visits (e.g., every 3–6 months). These tools can help identify cognitive deficits early, track changes over time, and inform treatment planning. For treatment, our study highlights the potential of cognitive interventions, which we briefly mentioned earlier in the manuscript as an effective approach for these disorders. Specifically, cognitive remediation therapy (CRT), a structured intervention involving exercises to improve attention, memory, and executive functioning, has shown promise in SCZ and BDP (45) . We suggest that clinicians incorporate CRT into inpatient and outpatient management protocols alongside OT.“

**3. In terms of the limitations, I appreciate that the authors acknowledge that the study’s relatively small sample size is a limitation. However, the authors do not address that the majority of study participants identify as male, which also limits generalizability. I find myself looking to the context to help explain why the sample mostly identifies as male, but little context is provided.**

We thank the reviewer for highlighting the potential for sampling bias and limits to generalizability in our study, as well as the absence of a control group as a limitation. These points have been addressed in the revised Limitations section of the Discussion, where we note the impact of the COVID-19 context (e.g., potential bias toward more severe presentations due to delayed care), the focus on hospitalized patients (limiting generalizability to outpatient settings), and the lack of a control group (preventing assessment of whether improvements were treatment-specific). Regarding potential sampling bias related to participant characteristics, we hypothesize that gender may have influenced our sample’s composition and outcomes. Specifically, in the context of Iran, cultural and social factors—such as stigma around mental health and traditional gender roles—may lead men to delay seeking treatment until their symptoms are more severe, potentially resulting in poorer clinical presentations at admission (e.g., higher PANSS scores) and less favorable treatment outcomes (e.g., smaller improvements in CDT or CGI scores). For instance, men in Iran may face greater societal pressure to maintain independence and avoid acknowledging mental health issues, which could delay hospitalization until a crisis point, as supported by literature on gender differences in mental health treatment-seeking behavior in Middle Eastern populations . In our sample of 84 participants, 58 (69%) were male, which may reflect this bias toward men presenting with more acute symptoms necessitating inpatient care. However, we did not conduct a formal analysis of gender differences in baseline severity or treatment response due to the study’s primary focus on diagnostic group comparisons. This represents a potential source of sampling bias that could have influenced the observed outcomes, particularly if men’s delayed treatment-seeking led to more severe baseline deficits. We have added this consideration to the Limitations section and recommend that future studies examine gender differences in presentation, treatment access, and outcomes in schizophrenia and BDP to better understand their impact on study findings and generalizability.

**4. Perhaps authors could comment on potential sampling bias and limits to generalizability with regard to collecting data during COVID-19 and to collecting data from study participants who were hospitalized. Another potential limitation may be that the study lacked a control group.**

We thank the reviewer for highlighting the limitations of our study . We have rewritten it and raised all important limitations:

“This study has several limitations that should be considered when interpreting the results. First, the sample size was relatively small (n = 42 per group), which may limit the generalizability of the findings. A larger sample could better detect subtle cognitive differences between groups. Second, this was a cross-sectional study assessing patients only at admission and discharge. A longitudinal design with extended follow-up could better characterize long-term cognitive and clinical trajectories. Third, cognitive assessment relied solely on the CDT, which, while sensitive, may not capture the full spectrum of cognitive domains; additional neurocognitive tests (e.g., assessing memory, attention) could provide a more comprehensive evaluation. Fourth, the lack of healthy controls prevented us from determining whether cognitive performance normalized with treatment. Fifth, we did not systematically record hospitalization duration for all participants, retrieving data for only a subset of 25 patients (mean = 21.28 days), which precluded analysis of its impact on outcomes; longer stays could theoretically enhance treatment exposure and influence recovery. Sixth, the study was conducted during the COVID-19 pandemic (September 2021 to December 2022), and while we included only COVID-19-negative patients, the broader context (e.g., delayed care, hospital constraints) may have influenced symptom severity at admission (e.g., high baseline PANSS scores). Seventh, the lack of detailed medication data (dosages, adherence) limited our ability to assess treatment effects on outcomes, particularly given the unreliability of pre-admission self-reports in this population. Eights,we did not explore potential gender differences in presentation or outcomes, though we hypothesize that men may present with poorer outcomes due to social or cultural factors affecting treatment-seeking behavior in Iran; this warrants further investigation in future studies .Larger, multi-center studies with longitudinal follow-up, comprehensive neurocognitive batteries, detailed treatment data, and control groups are warranted to confirm and extend these findings. Despite these limitations, this study provides preliminary evidence of distinct cognitive and clinical recovery trajectories in schizophrenia and BDP, emphasizing the importance of regular cognitive assessment in their management.”

We sincerely would like to thank the reviewers again for the valuable time and insightful comments on our manuscript. We appreciate the opportunity to revise and improve our work based on the constructive feedback we have received.
